# Improved Recognition of the Nutrition and Health Benefits of Nuts and Seeds Within the Health Star Rating System

**DOI:** 10.3390/nu17071195

**Published:** 2025-03-29

**Authors:** Véronique Braesco, Matthieu Maillot, Lise Becqueriaux, Sara Grafenauer

**Affiliations:** 1 VAB-Nutrition, 63230 Bromont-Lamothe, France; 2 MS-Nutrition, 13385 Marseille cedex 05, France; matthieu.maillot@ms-nutrition.com; 3 Bell Institute of Health and Nutrition, General Mills, Minneapolis, MN 55427, USA; lise.becqueriaux@genmills.com; 4 Faculty of Medicine and Health, School of Health Sciences, University of New South Wales, Randwick 2033, Australia; s.grafenauer@unsw.edu.au

**Keywords:** health star rating, front-of-pack labelling, nutrient profile model, health outcomes

## Abstract

**Background:** The health benefits associated with the consumption of nuts and seeds are well established, yet this food group is known to be the furthest from the recommended intake; therefore, actions aiming to increase nut intake are needed. The main front-of-pack communication device in Australia, the Health Star Rating (HSR), inadvertently penalises nuts with negative points associated with energy and saturated fat content. **Methods:** This study aims to suggest options to policy makers by (i) examining how the HSR rates a sample of 82 nuts, seeds and products containing them and (ii) testing three sets of moderate adjustments of the HSR algorithm on the sampled products: discounting the energy from nuts and seeds (S1), discounting the saturated fat from nuts and seeds (S2) and applying an adapted algorithm based on that for ‘oils and spreads’ for foods with ≥50% nuts and seeds (S3). **Results:** All three scenarios improved the Spearman correlation between the HSR score and the nut and seed content (−0.80, −0.75 and −0.71 for S1, S2 and S3, respectively) compared to the original HSR (−0.66). Products with more than 50% of their weight being nuts and seeds benefited much more from these adjustments than those below 50%. For all scenarios, but most clearly for S3, the products that had a lower HSR score than the original HSR (the healthier products) benefited more from the changes brought about by the adjusted algorithms than those of lower nutritional quality. The HSR of foods that contained no nuts or seeds remained unchanged. **Conclusions:** With minor changes to the HSR algorithm, nut and seed products could be brought into alignment with the current evidence, encouraging their regular inclusion in dietary patterns, which could help guide consumers at the supermarket shelf.

## 1. Introduction

There is ample evidence of the health benefits associated with high intakes of nuts and seeds. A recent review, synthesising around 90 studies, concluded that compared with no intake, a daily consumption of 28 g of nuts and seeds was associated with a reduction in the relative risk of cardiovascular disease by 21%, of cancer mortality by 11% and of all-cause mortality by 22%. The intake of nuts and seeds was also inversely associated with mortality from respiratory diseases, infectious diseases and diabetes [1]. A systematic analysis of the Global Burden of Disease (GBD) study, based on data collected worldwide over 36 years, revealed that diets low in nuts and seeds were associated with over 2 million deaths yearly, mostly from cardiovascular causes. The optimal range of intakes was calculated to be 16 to 25 g of nuts and seeds per day [2].

Aside from this large number of observational studies, often meta-analysed [3,4], other evidence, reviewed in Braesco et al. [5], comes from randomised controlled trials, demonstrating that the incidence of major cardiovascular events was lower among persons at high cardiovascular risk assigned to a Mediterranean diet supplemented with nuts than among those assigned to a reduced-fat diet [6] and documenting an improvement in blood lipids and blood pressure when consuming nuts [7]. The rationale for this wide range of beneficial health effects includes the high nutrient density of nuts and seeds, which contain mono- or polyunsaturated fats together with low levels of saturated fats and high levels of proteins, dietary fibre, vitamins (tocopherols, folates) and minerals (calcium, magnesium) [8,9].

The position of nuts and seeds in food-based dietary guidelines (FBDGs) varies between countries. They are not clearly depicted in 28% of the 90 countries that have national FBDGs [10], they are included in the fat food group in 23% of countries and they are within the protein-rich food group in 36% of countries [10], including the USA, Australia and New Zealand. In addition, they are considered a separate food group in France and Belgium [11]. Most countries recommend including nuts and seeds to diets, and quantitative recommendations, when they exist, recommend “a handful”, or amounts from 15 to 30 g, daily [10]. In Australia and New Zealand, there is no precise recommended intake for nuts and seeds: two to three servings are recommended daily from the protein group, to which nuts and seeds belong, and a serving size of nuts and seeds is set at 30 g or a “handful” [12,13]. However, no recommendation is given concerning the balance of foods that should contribute to these protein servings.

The intakes of nuts and seeds are low, and often very low, in all countries. In the aforementioned GBD study, the mean daily intake of nuts and seeds was only 3 g, the food group furthest from its optimal intake [2]. In Australia, in 2013, the mean nut intake was 4.61 g/d, with only 5.6% of nut consumers consuming 30 g of nuts per day [14]; around two-thirds of nuts were consumed in between meals during snacking occasions [15]. In New Zealand, the mean intake of nuts was 5.2 g/day, with 29% of adults considered consumers, indicating that more than 70% were not consuming any nuts or seeds [16]. Based on published data [1], modelling suggests that increasing nut consumption among Australians from the current intake of 4.6 g to 30 g per day would contribute to an estimated reduction in health care expenditures of at least AUD 980 million per year [17]. There is a need for efficient policy measures aimed at increasing intakes of nuts and seeds.

Among such measures, front-of-pack nutrition labels (FOPNLs) are increasingly being used to inform consumers about the nutritional value of food products. The Health Star Rating (HSR) system, adopted in 2014 by the Food Standard Agency of Australia and New Zealand (FSANZ), is an interpretative system that ranks foods. It is based on an algorithm that considers nutrients to limit, including energy, saturated fat (SFA), sodium and sugar, and components to encourage, such as dietary fibre, proteins and fruits, vegetables, legumes and nuts. The resulting score is then translated into stars (0.5 to 5), conveying an easy-to-understand message [18]. An analysis of over 47,000 Australian packaged foods revealed that the median star numbers were 4.0 (3.0 to 4.5) for core products and 2.0 (1.0 to 3.0) for discretionary products [19], and a score ≥ 3.5 was generally accepted as an indication of healthier products [20]. In spite of a good level of awareness and trust among consumers [21] and the conclusion that the system performs well following the review after five years of implementation [22], some room for improvements remains. For example, it has been argued that the HSR algorithm may not adequately communicate the benefits for consumers of swapping to whole grain foods; many refined and whole grain products have the same number of stars despite the clear benefits of the whole grain varieties [23]. This may also be the case for nuts and seeds; indeed, although nuts are included in the positive component, this may not be sufficient to overrule the negative points associated with their content of energy and saturated fats, resulting in a penalisation of nut-containing foods rather than incentivising consumption. For example, using data from the FSANZ food composition tables, the HSR would currently rate a whole meal bread with 20% walnuts included 4 stars, 0.5 stars lower than a plain whole meal bread.

As for other FOPNLs, such as the French Nutri-Score, displaying the HSR on food products remains voluntary. However, recently, the Australia and New Zealand governments expressed their disappointment, as implementation is significantly off-track and meeting the target of 70% of products voluntarily displaying their HSR by the end of 2025 is unlikely [24]. There are calls for the HSR to become mandatory, and therefore, it is timely to recommend slight adjustments in its algorithm to ensure these are consistent with both the latest scientific literature and the messages and evolutions of dietary guidelines. It would therefore be opportune for authorities to consider possible enhancements in the near future [25]. Indeed, improving the system and solving its current limitations would incentivise improved consumer food choice behaviour and manufacturer reformulations while authorities consider the transition towards the HSR being a mandatory feature on packaging.

A relevant parallel is the situation in Europe, where a mandatory harmonised FOPNL was announced by the European Commission [26]. The Nutri-Score, a system developed in France and adopted by several European countries on a voluntary basis, is a candidate to become the European FOPNL. Its algorithm was recently updated to better align with FBDGs. For example, the scoring of fatty fish, rich in long-chain polyunsaturated fats beneficial to health, was improved; this was also the case for some vegetable oils, particularly the more favourable choices (canola, olive, nut oils…), due to their rich polyunsaturated fatty acid content and links with better health outcomes. As part of this revision, nuts have been moved from the ‘fruit and vegetable’ component to the ‘fats, oils, nuts and seeds’ component, with associated changes in the algorithm [27] allowing for a score that better reflects the health value of nuts and nut-containing foods.

In this context, this study aims to (i) examine how the current HSR algorithm rates a sample of typical nuts, seeds and nut- or seed-containing products, (ii) test scenarios consisting of moderate adjustments of the HSR algorithm for plain nuts or seeds and nut- or seed-containing products to better recognise their nut or seed content and (iii) provide suggestions and data helpful for decision-makers when the HSR is next reviewed.

## 2. Materials and Methods

### 2.1. The Health Star Rating (HSR) System

The HSR is a FOPNL based on the nutrient and ingredient contents of packaged foods, as detailed in [28]. Briefly, the algorithm considers four components associated with increasing the risk factors for chronic diseases: energy, saturated fat, sodium and total sugars. It also considers certain ‘positive’ components of a product such as content of dietary fibre, fruits and vegetables, nuts, legumes and, in some instances, protein. Taking these components into account, points are allocated based on 100 g or 100 mL of the product and the resulting score can in theory vary from −38 (best nutritional quality) to +96 (worst one). The score is then translated into stars, whose attribution depends on thresholds that vary according to food categories, as defined in the HSR guide [28]. For example, 0.5 stars (flagging products with the lowest nutritional quality) are attributed when the score is 42 or above for ‘oils and spreads’ (HSR category 3). For all other foods except dairy, beverages and jellies (HSR category 2), 0.5 stars are attributed when the score is above 25. Similarly, the maximum number of stars (five) is attributed to ‘oils and spreads’ when the score is plus 13 or below, but the score for other foods should be minus 11 or below to be translated into five stars. The system is thus less demanding for ‘oils and spreads’ than for other foods, as illustrated in Figure S1.

### 2.2. Three Scenarios Developed for Adjusting the HSR Algorithm

#### 2.2.1. Scenario S1: Discounting the Energy Content of Nuts

This scenario discounts the energy content of nuts and seeds in the energy component of the HSR algorithm. The energy in nuts and seeds largely comes from fats, and discounting this nutrient source is an alternative way of considering the overall favourable quality of the fat in nuts and seeds; furthermore, there is consistent evidence that unlike the energy from most foods, the metabolizable energy from nuts is less determined by food chemistry measurements, as predicted by Atwater factors [29,30,31].

#### 2.2.2. Scenario S2: Discounting the SFA Content of Nuts

Scenario 2 discounts the SFA content of nuts and seeds within the SFA component of the HSR algorithm. Indeed, SFAs in nuts and seeds are accompanied by larger amounts of mono- and polyunsaturated fatty acids [5] and should not be considered in the same way as isolated SFAs from other sources, which do not bring nutritional benefits. For each food, the value used to calculate the number of points associated with the SFA element corresponded to the SFA content from the different nuts and seeds in the product (calculated using the information available in the Australian food composition table [32] subtracted from the total SFA content).

#### 2.2.3. Scenario S3: Replacing Energy with Energy from SFAs Only and SFAs with Ratio of SFAs/Total Fat

This scenario was built to test the changes that were recently adopted by the French Nutri-Score when its scientific committee undertook an in-depth revision intended for a better alignment with FBDGs. Among other considerations, it was estimated that nuts had a favourable nutrient profile and must be placed, together with seeds, in the same category as their respective oils to avoid discrepancies [27].

In this third scenario, only food products containing 50% nuts and seeds or more in weight were concerned by the modification. Products with fewer nuts and seeds were evaluated using the original HSR without any change.

For the concerned products, two changes to the original HSR algorithm were applied: firstly, the energy component of the HSR was replaced by the energy from SFAs only, encouraging the high proportion of unsaturated fats for which the energy was not accounted for. This led to changes in applying score points, and the Nutri-Score scale was used but adapted by adding an additional cut-off, set at 60 kJ, to keep the 11-point scale of the HSR instead of the 10-point scale of the Nutri-Score (Table S1). Secondly, SFAs were replaced by the ratio of SFA/total fat (in %). However, the Nutri-Score scale for this component comprises only 10 points, and intermediate cut-offs were added to maintain the 30 points of the initial HSR scale (Table S1).

In this scenario S3, the thresholds used to translate scores into numbers of stars were those used for HSR category 2 products (i.e., all foods but oils, spreads, dairy and jellies) and not the thresholds applied for oils and spreads (HSR category 3). This approach was chosen to avoid attributing too many stars to products with high HSR scores (see above). For illustrative purposes, some attempts were, however, made to use category 3 thresholds.

### 2.3. Selection of Food Products to Test the Three Scenarios

A database of the nuts, seeds and nut- and/or seed-containing products sold on the Australian and/or New Zealand markets was established in order to reflect the variability in nut/seed content across nuts, seeds and nut- or seed-containing products on the market.

For plain nuts and plain seeds, data were collected from the FSANZ food composition table [32]. Manufactured products containing nuts and seeds were identified from the websites of the major retailers in these countries, as well as Australian and New Zealand versions of other manufacturers’ websites, visited in June and July 2024. To avoid complexity, only food products that fit in the category 2 of the HSR were selected, and no product that would fall into another version of the algorithm was included. This excluded beverages, dairy foods and nut-based dairy alternatives. Except for these, all groups of manufactured foods that contained nuts or seeds were explored. Individual foods were grouped according to the market category to which they best corresponded, i.e., bread, breakfast cereals, cakes, cookies, bars (also known as ‘muesli’ or ‘cereal’ bars), cookies, ice creams, butters and spreads, mixed and coated/salted nuts or confectionary (chocolate and non-chocolate).

Only products for which the necessary information was available (nutrient and ingredient contents, as required by the original HSR [28], plus the nature and weight percentage of nuts and seeds) were included. The resulting database was not intended to be representative or exhaustive.

A control sample of foods that did not contain nuts or seeds was also collated, aiming at assessing the potential side effects of each scenario on the HSR using a variety of commonly consumed products.

### 2.4. Calculations and Statistical Analyses

The original HSR algorithm and the three scenarios were applied to the overall sample (82 products containing nuts and seeds and 9 products without nuts or seeds). Stars were attributed using the thresholds for HSR category 2, for the original HSR and for scenario S3, and calculations were also made using the thresholds of HSR category 3. Calculations utilising the nine non-nut products were carried out to check that the modifications did not impact the HSR score for these products.

All statistical analyses comparing the original HSR against the three scenarios were conducted on the sample of 82 products containing nuts and seeds.

The means of the HSR scores (original HSR and scenarios S1, S2, S3) and means of the proportions of nuts and seeds were also estimated by food type. The distribution of the HSR scores were represented using a boxplot for the whole sample and separately for products with nuts and seeds below or at/above 50%. For each scenario, a Wilcoxon–Mann–Whitney test was used to compare the median HSR score of products with 50% or more of nuts and seeds against those with less. This threshold of 50% was arbitrarily retained following the latest revision of the French Nutri-Score [27], which applies scenario 3 to products containing more than 50% nuts.

The Spearman coefficient correlation was estimated between each HSR score and the proportion of nuts and seeds among the whole sample, as well as separately for products with nuts and seeds above or below 50%; the correlations when using the scenarios S1, S2 and S3 were statistically compared to the correlation using the original HSR via Fisher’s Z-transformation for paired samples.

The number of products in each HSR star range (from 0.5 to 5) was calculated and compared between all HSR algorithms (original and the three scenarios). The proportion of products having a star rating of 3.5 or more in scenarios S1, S2 and S3 was statistically compared to the proportion in the original HSR following a McNemar’s test.

Each HSR score (for S1, S2 and S3) was plotted against the original HSR by grouping products according to their proportion of nuts and seeds (below or above 50%) to visualise products with an improvement in their score. The percentage of products with an improvement (or deterioration) in their HSR star number was estimated for each scenario on the whole sample and according to their proportion of nuts and seeds. The variations in the S1, S2 and S3 scores with the S0 score were statistically compared using a Kruskal–Wallis test. All analyses and plots were carried out using SAS software 9.4. All *p*-values below 5% were considered significant.

## 3. Results

### 3.1. Selection of Nuts, Seeds and Nut- or Seed-Containing Products

A total of 82 food products containing from 1 to 100% nuts and seeds, as labelled in the ingredient list, were included in the assessment. These foods belonged to 12 different food types. The five most represented were “bars (also called « muesli bars » in Australia and New Zealand)” (n = 15; 18%), “plain nuts” (n = 13; 16%), “mixed and coated/salted nuts” (n = 9; 11%), “breakfast cereals” and “nut butters” (each n = 8; 10%). The mean nut and seed content was 53.8 ± 38.6%. For each food type, the number of products included in the database and the mean nut and seed contents are provided in Table 1. Detailed compositional information of each product is available in Supplementary Table S2, which also contains information on the control products that did not contain nuts or seeds.

### 3.2. Rating of Selected Foods in the Initial HSR Profiling System

When using the current HSR algorithm, the mean score of the assessed products was 3.30 ± 14.54, and the star number varied from 0.5 to 5. Table 1 displays the HSR score for each food type, and the HSR score and star number of each product are available in Table S3. Caution is needed when interpreting data for food types that contain only a few products, such as cakes or ice creams. Regarding the 13 plain nuts included in the list, 5 (38%) were given a score corresponding to five stars and 2 (Brazil nut and macadamia nut) were granted four stars. This means that not all plain nuts would be consistently labelled with five stars. Coconut, considered as a nut in the HSR system [28], was rated with 3 stars for the fresh flesh of the mature fruit and 2.5 when grated or desiccated. All plain seeds were rated five stars.

**Figure 1:**
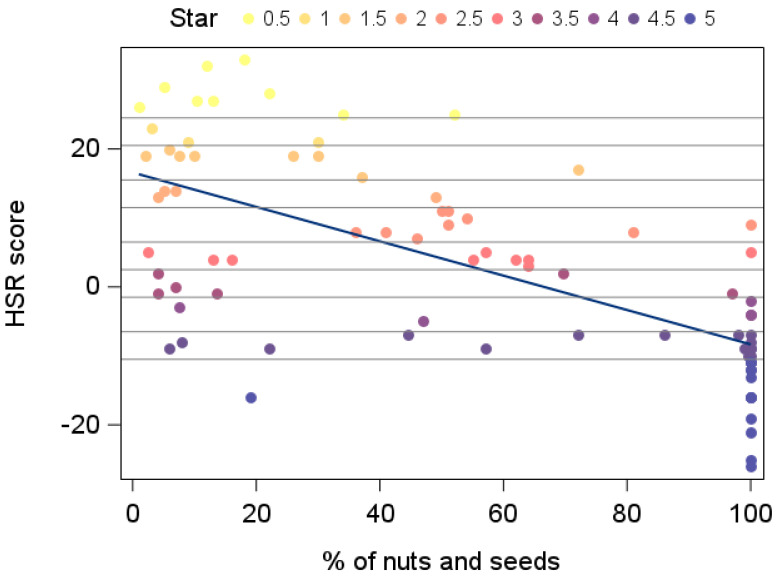
Association of the original HSR score with the content of nuts and seeds (weight %) of 82 food products. The regression equation reads Y = 16.58 − 0.25X. The horizontal reference lines refer to the HSR score thresholds used to determine the number of stars.

The correlation between the proportion of nuts and seeds (weight percentage) within each food and their HSR score was moderate (Spearman correlation: −0.66) (Figure 1) when calculated over the whole range of nut- and seed-containing products. When the correlation was calculated separately for products with a nut and seed proportion above and below 50%, a weaker correlation was found for products containing less than 50% nuts and seeds (Spearman correlation −0.10) compared with a stronger correlation for products containing 50% or more nuts (Spearman correlation: −0.72), indicating that the correlation is mainly driven by the products high in nuts.

### 3.3. Application of the Three Scenarios to the Selected Food Products

#### 3.3.1. Scores and Stars

The mean HSR scores when the various scenarios were applied were −1.37 ± 16.09 for scenario S1, −1.27 ± 16.49 for scenario S2 and 0.48 ± 17.11 for scenario S3, to be compared to 3.30 ± 14.54 for the original HSR algorithm. The scores by food type are displayed in Table 1, and the full data set for each product is available in Table S3.

**Figure 2:**
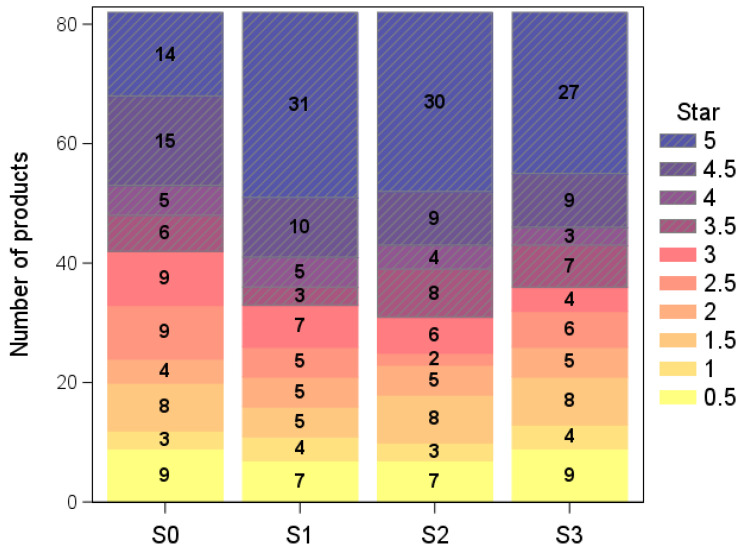
Number of nut- and seed-containing products that bear each number of HSR stars based on category 2 thresholds (by half-units from 0.5 to 5) according to original HSR (S0) and scenarios S1, S2 and S3.

When the HSR scores were translated into stars according to the thresholds associated with products of HSR category 2 in the original HSR guide (i.e., all products but dairy, beverages, and oils and spreads [28]), applying the alternative scenarios resulted in an overall slight increase in the number of products bearing a higher star rating, which was broadly similar for the three scenarios (Figure 2). When focusing on the products with ≥3.5 stars, the threshold that signals a healthier nutrient profile, scenario S1 resulted in 60% of products bearing 3.5 stars or more, and this percentage was 62% and 56% for scenarios S2 and S3, respectively, as compared with 49% for the original HSR (all statistical comparisons were significant, *p*-value = 0.0027; <0.001; 0.0143 for scenarios S1, S2 and S3, respectively).

The rationale behind scenario S3 involved considering products with 50% nuts and seeds or more as ‘oils and spreads’; therefore, the number of stars was also estimated for scenario S3 by using the thresholds in the original HSR for ‘oils and spreads’ (HSR category 3 [28]). As these thresholds were largely more favourable (i.e., they assigned a higher number of stars for a same score than when using the HSR category 2 thresholds), this resulted in a high proportion of food products with an elevated number of stars. In particular, 51% of products were rated five stars, and only 18%, 39%, 37% and 34% were rated as such for the original HSR and scenarios S1, S2 and S3, respectively, when using the category 2 thresholds. This exemplifies the strong impact of the thresholds set for translating HSR scores into stars. Indeed, when category 3 thresholds were applied to the scores obtained with the original HSR, the number of products bearing five stars was 52% (43 products), compared with 18% when category 2 thresholds were applied (Figure S2).

Whatever the scenario or the category thresholds, no scores and thus no star numbers were changed for the non-nut-containing products.

#### 3.3.2. Influence of Product Characteristics on HSR Score and Stars

The nut and seed content affects the scores, and thus the stars, of the food products in all scenarios, including the original HSR. The Spearman correlations between the percentage of nuts and seeds and the HSR score significantly improved in scenarios S1, S2 and S3 (−0.80, *p*-value < 0.001; −0.75, *p*-value < 0.001; and −0.71, *p* value = 0.044, respectively) compared with the original HSR (−0.66).

**Figure 3:**
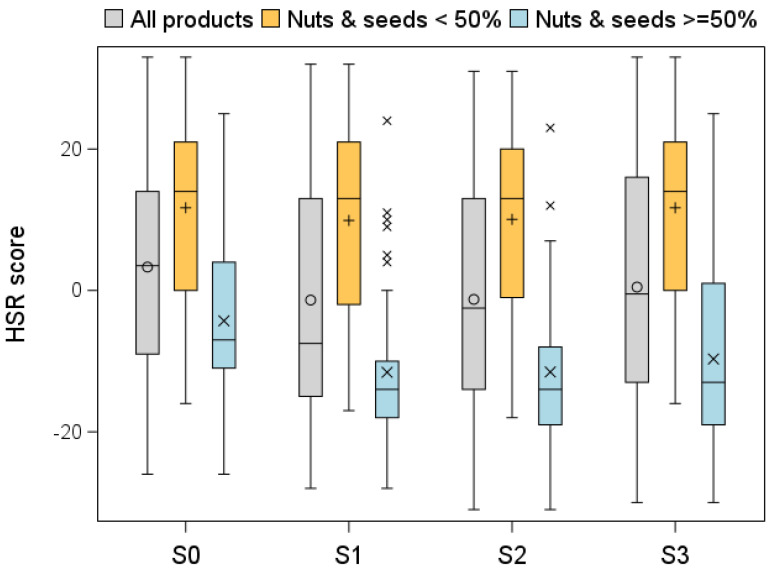
Box plot of HSR scores according to the original HSR and the three scenarios for all food products, products containing 50% or more nuts and seeds and those containing less than 50% nuts and seeds. The circles and crosses within the boxes indicate the mean values. The whiskers are drawn from the box to the most extreme point that is less than or equal to 1.5 times the interquartile range (third quartile minus first quartile). The crosses outside the box show products with an HSR score higher than the upper whisker.

This resulted in differences between products that contained more than 50% nuts and seeds vs. those that contained less, both in the original HSR and in the three scenarios (Figure 3). The difference between the products with more vs. less than 50% nuts and seeds was 15.99 score points using the original HSR algorithm, whereas it exceeded 21 points when using any of the three scenarios (21.48, 21.58 and 21.39 points in scenarios S1, S2 and S3, respectively). The score difference between the products with more vs. less than 50% nuts and seeds was statistically different in each scenario (*p*-value 0.0002 for original HSR; *p*-value less than 0.0001 for each of scenarios S1, S2 and S3).

Analyses of the number of stars confirm the findings produced from the associated scores. For products that contained less than 50% nuts and seeds, the star ratings were not affected by any scenario, with 28%, 31%, 33% and 28% of products exceeding three stars in the original HSR and in HSR scenarios S1, S2 and S3, respectively. However, among products with 50% or more nuts and seeds, 36% were above three stars using the original HSR, while this was the case for 46%, 47% and 45% under scenarios S1, S2 and S3, respectively. The *p*-values for comparisons with the original HSR were 0.0047; 0.0027; and 0.0143 for scenarios S1, S2 and S3, respectively.

While the mean increase in the star numbers never exceeded one star, this varied according to food type. Table S4 shows that the mean number of stars was not increased for some types, particularly those considered discretionary foods, such as cakes and ice creams. For confectionary products, the half-star increase for some scenarios corresponded to a higher nut and seed content, such as nougat, or nutty bites, which contained 50% nuts or more. Regarding cookies, the same increase in the mean number of stars was due to a single product, coconut macaroons, which contained 30% coconut (see Table S2 for details). There was an increase of one star only for the food types that contained the most nuts and seeds, such as plain nuts and bars with more than 50% nuts and seeds.

#### 3.3.3. Changes in HSR Scores and Star Numbers Between the Three Scenarios

**Figure 4:**
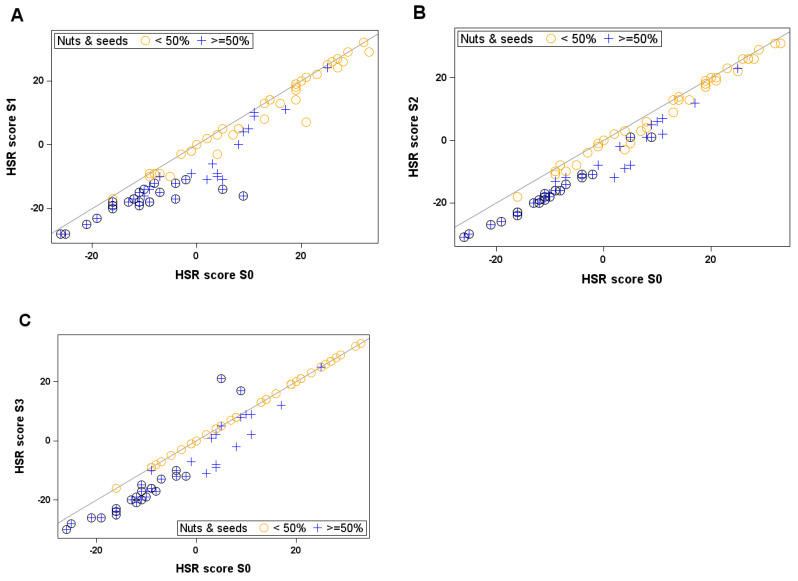
Switch of HSR score from original HSR to each scenario ((**A**): S1; (**B**): S2; (**C**): S3); each yellow circle represents a food product with less than 50% nuts and seeds, and each blue cross a product with 50% nuts and seeds or more. Products in a black circle are plain nuts and plain seeds (100% nuts or seeds) When product is on diagonal line, its score does not change compared to original HSR. When food is below line, its score is decreased, i.e., its nutritional quality is better rated compared to original HSR. When food is above line, its score is increased, i.e., its nutritional quality is rated worse compared to original HSR.

Compared with the original HSR, all the scenarios improved the HSR score (Table 1), with smaller changes brought by through the application of scenario S3 compared to S1 and S2 (Figure 4). Overall, 65 (79%), 71 (87%) and 39 (48%) of products improved their score by at least one point in scenario S1, S2 and S3, respectively, when compared to the original HSR. Mean improvements reached −4.67 points for S1, −4.57 points for S2 and −2.83 points for S3. The HSR score changes compared with the original HSR were different for scenarios S1 and S2 (*p* value 0.023 and 0.037 for scenarios S1 and S2, respectively), but not for scenario S3. Only two products (2.4% of nut- and seed-containing products in the sample studied), both containing a high proportion of coconut, saw their score increased (reflecting a lower rating of their nutritional quality), both only in scenario S3 (Table S3).

When only products with less than 50% nuts were considered, improvements when compared to the original HSR were not statistically significant and were seen in a smaller proportion of products, i.e., 58% and 72% of products in scenarios S1 and S2, respectively, and no score was improved under scenario S3. The magnitude of score improvements within a scenario was also lower when products contained fewer nuts. For example, in scenario S1, the mean score improvement was −1.79 in the 39 products containing less than 50% nuts, compared to −7.28 for those with more than 50% nuts (Table S5).

Conversely, when only products containing 50% or more nuts and seeds were evaluated, the changes in score when compared with the original HSR scores were highly significant for all scenarios (*p* value = 0.0004, 0.0013 and 0.0098 for scenarios S1, S2 and S3, respectively).

The magnitude of the change in rating varied according to the initial HSR score. For example, the score of the hazelnut chocolate spread (27 points in the original HSR) was not affected by the three scenarios, whereas one of the natural almond spread products (−16 points in the original HSR) was decreased by four, seven and eight points in scenarios S1, S2 and S3, respectively (Table S3).

Food products of better nutritional quality (3.5 stars or more according to the original HSR) saw their HSR score improved (i.e., indicating an even better nutritional quality) more than products of lower nutritional quality (3 stars or less according to the original HSR). In scenario 2, the improvement was of −3.69 and −5.50 in foods of lower and higher nutritional quality, respectively (*p*-value: 0.0002), and for scenario 3, the improvement was of −0.79 and −4.98 in foods of lower and higher nutritional quality, respectively (*p*-value < 0.0001). In scenario 1, there was no significant difference in score change according to the nutritional quality of the food.

Improvements in HSR scores logically resulted in improvements in the number of stars. Over the 82 food products of the database and relative to the original HSR, scenario S1 led to a mean increase of 0.4 stars (min 0; max 2.5; *p*-value 0.0040), scenario S2 to a mean increase of 0.37 stars (min 0; max 1.5; *p*-value 0.0018) and scenario S3 to a mean increase of 0.17 (min 0; max 1.5; *p*-value < 0.0001).

**Figure 5:**
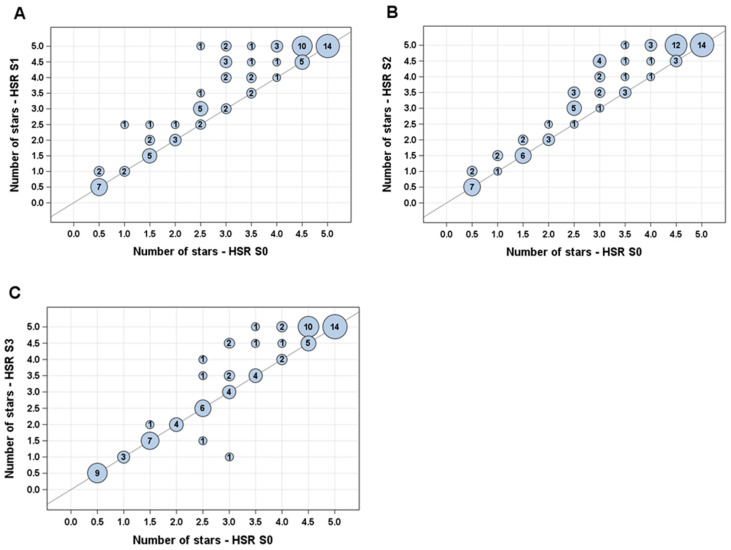
Switch in star number from original HSR to each scenario ((**A**): S1; (**B**): S2; (**C**): S3). Bubbles on diagonal line show number of products for which star number did not change. Bubbles above line show number of products for which number of stars increased. The larger the bubble, the higher the number of concerned products.

Figure 5 displays the change in the number of stars from the original HSR to each scenario. Increases in the number of stars were more frequent for products that initially had a higher number of stars, suggesting that products of a lower nutritional quality did not, or very minimally did, improve their star rating. This was, however, only significant for scenario 3, for which 16.7% of products saw their star ratings increased if they scored fewer than three stars with the original HSR, whereas this was the case for 37.5% of the products if they had three stars or more under the original HSR algorithm.

The variation in the number of stars varied also with food types and with the nut or seed content of the products (Table S3). Here, again, scenario S3 resulted in smaller effects than scenarios S1 and S2; indeed, 31%, 33% and 0% of the products containing less than 50% nuts and seeds saw their star number increase after applying scenarios S1, S2 and S3, whereas this was the case for 61%, 66% and 50% of the products containing more than 50% nuts (Table S6).

## 4. Discussion

In the context of the significant gaps between recommendations and actual intakes, FOPNLs are one way to help guide consumers to increase their intake of nuts and seeds by flagging products that contain higher amounts of nuts and seeds and by encouraging manufacturers to include more nuts and seeds in their products. This premise assumes that the algorithm supporting each FOPNL appropriately reflects the nutritional value of nuts and seeds.

Although the score given by the original HSR was correlated with the content of nuts and seeds within products, this correlation remained moderate, and slight adjustments in the algorithm may result in more rewarding scores, and thus in more stars for products that contain more nuts and seeds. The purpose of this study was to propose and test some of the possible adjustments that better align with nut and seed content so this can be better acknowledged by the HSR scores and stars.

The results show that products with more than 50% of their weight comprising nuts and seeds benefit much more from these adjustments than those below 50%, avoiding undesirable rewards for products with a lower nut and seed content. This is important to allay concerns regarding misalignment with public health policies. Although arbitrarily set, this 50% threshold is practically meaningful, as it helps in showing that these improvements primarily concern products with a significant proportion of nuts and seeds. Compared to the original HSR, the proposed adjustments also allowed the algorithm to better discriminate products according to their content of nuts and seeds, which is of major importance to help consumers increase their intake of nuts and seeds. Indeed, consumers understand and use the HSR star logo [19], and it can be expected that this is reflected in their purchases. The amendments we suggest especially improve the number of stars of products that contain 50% or more nuts and seeds, and this may encourage consumption. For example, a 30 g bar containing 55% nuts with 3 stars would increase to 4.5 and would provide a significant additional intake of 16 g of nuts. Similarly, and in accordance with FBDGs that do not make distinctions between different nuts, five stars will be affected to all plain nuts, facilitating their more frequent selection as “healthy foods”.

Importantly, all of the tested scenarios led all plain nuts and seeds to be awarded five stars and thus to be considered among the healthiest foods. This is important as differences of 0.5 stars between some types of nuts, as observed under the original HSR, are potentially confusing for consumers, particularly as FBDGs do not distinguish between different nut varieties. As all plain nuts are natural products that cannot be reformulated, these should be automatically awarded five stars, which is made possible by the three scenarios presented, conveying a much clearer message to consumers.

Manufactured products that contain high amounts of nuts and seeds (nut spreads, bars...) are also better acknowledged within all of the scenarios. However, those with a medium to low nutritional quality did not dramatically increase their star rating, which is reassuring from a public health point of view. Indeed, the nutrients to limit that can come with nuts and seeds in discretionary products remain duly taken into account and penalised by the algorithm in the proposed scenarios. Within the tested database, most nut-containing discretionary foods were not influenced by the changes, and only a few earned. We will a half to one more star with the proposed adjustments; this was the case for nougat, a confectionary that contains 50% almonds and that increased from 2.5 to 3.5 stars, favouring a better discrimination vs. other confectionaries without nuts and of poorer nutritional value.

Our analysis showed that, especially for scenario 3, the products that had a lower HSR score and a higher star rating with the original HSR (the healthier products) benefited more from the changes brought by the adjusted algorithms than those of lower nutritional quality. Also, the changes were of a greater magnitude when products contained more than 50% nuts and seeds, and scenario S3 did not, by definition, affect the rating of products with less than 50% nuts and seeds.

Questions may be raised about the potential different health effects of nuts and seeds depending on whether they are consumed as whole food items or incorporated into other foods. The forms under which nuts and seeds are considered in studies that support the health effects of nuts are probably different from one study to another. Across the numerous observational, whole nuts, and seeds (including plain, savoury, and sweetened nuts) and nut-based butters and spreads are always included while a few studies may also include nut- or seed-containing foods [3,33,34]. In intervention trials, as meta-analysed in [35], most interventions provided whole nuts, while some found favourable effects on body weight management and of the cardio-vascular parameters of nuts incorporated into cereal bars [36,37] or in meat products [38]. Overall, whatever the form under which they are consumed, there is a large consensus on the health benefits associated with intakes of nuts and seeds at levels that largely exceed current consumption.

One drawback was identified in relation to coconut; regardless of whether fresh or dry, coconut is considered a nut in the HSR guidelines [28] despite its nutritional characteristics being very different to other nuts. Indeed, coconut contains around five to ten times more saturated fats than other nuts, and as scenarios 2 and 3 change the way saturated fats from nuts were considered, this resulted in an unreasonable improvement in the score of products containing coconut. This questions the position of coconut within this classification.

The three proposed scenarios [discounting energy from nuts and seeds (S1), discounting saturated fats from nuts and seeds (S2) and treating foods with more than 50% nuts and seeds similarly to ‘oils and spreads’ in the original HSR (S3)] are of nutritional relevance: the high energy density of nuts has not been associated with weight gain [39] and their saturated fat content is offset by a high proportion of health-promoting unsaturated fats [8]. The last scenario (S3) was directly inspired by the recent change to the French Nutri-Score to take into account, among other factors, the specific nutritional composition of nuts [27]. All of the scenarios better reflected the nutritional value of nuts and seeds compared to the original HSR algorithm, but scenario S3 resulted in smaller improvements, both in scores and in stars, if HSR category 2 thresholds were used to assign stars. Attempts to use the thresholds for category 3 products would lead to most products with 50% or more nuts and seeds bearing five stars (Figure S1), a rather non-discriminant and undesirable outcome. If scenario 3 is to be utilised, products containing 50% or more of nuts and seeds should not be considered as “oils and spreads” (category 3 foods), as is the case with the Nutri-Score, or the category 3 thresholds for stars may need to be reconsidered to better discriminate between products with the greatest proportions of nuts and seeds.

This study has limitations. The product database was neither exhaustive nor representative of the nut- and seed-containing food products sold in Australia and New Zealand. We did not include a few rare nuts and seeds, such as hickory or sapucaya nuts and cotton or wattle seeds, or discarded chestnuts, whose nutritional composition and uses are very different from other nuts. Regarding composite products, completeness of the ingredient and nutrition composition was a pre-requisite for this study, and this led to the removal of products lacking the necessary information, most often regarding the nature and proportion of nuts and seeds in the product. This limitation prevented broad conclusions from being made regarding product types, and we acknowledge that careful interpretation is necessary given the small size of the food type samples. A confirmation of our findings using a more extensive database of nut-and seed-containing products should be considered. In particular, it should include the full range of nut-based beverages, substitutes and plant-based alternatives that were not featured in our database. However, this study’s objective was to demonstrate that slight adjustments to the HSR algorithm are feasible, favourable and reasonable, and this objective was met. Importantly, our suggested changes were validated through an examination of a range of non-nut/seed-containing products to ensure no unintended consequences of the changes explored.

Other scenarios to alter the HSR have been suggested; for example, the alpha-linolenic acid (ALA) content of nuts and seeds is of major interest [40] and could be considered for nutrient profiling adjustments. However, it would probably be practically difficult and costly for manufacturers to provide the ALA content of their products, as ALA is not usually reported. Additionally, integrating ALA would lead to profound changes in the HSR algorithm. Similar to the objectives of the present study, modifications on a scale of up to 10 points have been modelled for whole grain products containing 25–100% whole grain, with the preferred option adjusting the HSR score cut-off by 3 points, creating the greatest difference in median HSR between refined and whole grain items (up to two stars difference). The suggested change better aligns with an emphasis on dietary guidelines compared to the original algorithm, where many core grain foods are poorly differentiated [23,41].

A major strength of this analysis is that the fundamentals of the HSR algorithm were maintained within each of the three scenarios and the changes did not modify the structure or logic of the system. The scenarios were easily implemented, as not counting energy or SFAs from nuts and seeds is a minor adaptation, especially as the nutritional values of nuts are publicly available. Finally, it was ascertained as part of this analysis that the adjustments made to the HSR algorithm do not impact food products not containing nuts.

## 5. Conclusions

The nut and seed content of foods is rewarded through the HSR algorithm but inadvertently also receives negative points associated with their inherent energy and saturated fat content. Through this analysis, we propose refinements to the algorithm to better account for the nutritional features of nuts and seeds, which may be useful for FSANZ, who are now managing the HSR. As the suggested target of 70% of foods under voluntary labelling provisions by November 2025 is unlikely to be reached, the mandatory use of the HSR will be considered in the future. Ensuring that the HSR reflects the true value of foods will be increasingly important as the key communication tool for front-of-pack labelling for consumers. This cannot, however, be sufficient to fill the large gap between the recommendation for nut and seed intakes and their actual levels of consumption, and other policy measures remain needed.

Adjusting the HSR, rewarding nut and seed content in a similar manner, may have flow-on effects to manufacturers, incentivised to include nuts and seeds in their products. Importantly, it was the foods with the highest nut contents that benefited the most through our analysis, potentially sending a clearer message to consumers who may be unsure about including nuts and seeds to their diet due to their energy and fat contributions or who may be hesitating due weight management concerns. A range of manufactured food products would also benefit from the scenario proposed, but less so those considered discretionary food products with a lower nutritional value, an important outcome from a public health viewpoint.

Health professionals in Australia are well supported with information regarding nuts and their health benefits, but other government-driven materials have been slow to highlight the true benefits of nuts and seeds, and this includes dietary guidelines. We have demonstrated that through a minor change to the HSR algorithm, nut and seed products could be brought into alignment with current evidence, encouraging the regular inclusion of these foods in dietary patterns and helping guide consumers at the supermarket shelf.

## Figures and Tables

**Table 1:** Proportion of nuts and seeds and scores using original HSR and three scenarios by food type.

		Nuts and Seeds		% of Nuts and Seeds		Score Using Original HSR		Score Using Scenario S1		Score Using Scenario S2		Score Using Scenario S3	
	N	Nb ≥50 %	Nb < 50%	Mean	Std	Mean	Std	Mean	Std	Mean	Std	Mean	Std
All products	82	39	43	53.28	38.49	3.30	14.54	−1.37	16.09	−1.27	16.49	0.48	17.11
Food type													
*Breads*	3	0	3	9.67	8.14	−8.67	7.51	−9.33	7.51	−9.67	8.50	−8.67	7.51
*Breakfast cereals*	8	0	8	9.94	6.64	1.50	6.70	0.38	7.03	−0.75	6.76	1.50	6.70
*Cakes*	3	0	3	5.67	2.08	6.33	12.42	6.00	13.00	6.33	12.42	6.33	12.42
*Bars*	15	9	6	51.77	12.93	6.20	7.44	−1.13	9.64	−0.53	10.20	2.93	10.50
*Chocolate confectionary*	6	2	4	31.50	17.09	25.67	7.94	24.17	7.99	23.33	8.87	25.33	8.69
*Cookies*	5	0	5	16.10	12.93	20.20	1.79	16.60	5.68	18.80	2.49	20.20	1.79
*Ice creams*	5	0	5	5.66	4.12	22.60	3.65	22.00	2.92	22.20	3.49	22.60	3.65
*Non-chocolate confectionary*	2	1	1	27.50	31.82	20.00	12.73	19.50	13.44	15.50	19.09	15.50	19.09
*Nut butters*	8	6	2	76.44	39.45	−3.38	13.63	−8.50	16.03	−9.00	16.16	−8.75	16.75
*Nuts*	13	13	0	100.00	0.00	−7.15	7.51	−15.85	2.61	−14.54	7.86	−12.00	14.33
*Seeds*	5	5	0	100.00	0.00	−20.40	5.98	−24.40	4.16	−26.20	5.54	−25.00	5.83
*Mixed and coated/salted nuts*	9	7	2	78.06	22.39	−1.44	9.30	−7.67	8.66	−7.11	9.58	−6.22	9.42

## Data Availability

All raw data are available in the Supplementary Materials.

## Supplementary Materials

The following supporting information can be downloaded at https://www.mdpi.com/article/10.3390/nu17071195/s1, Figure S1: Thresholds used to assign stars to a food product, according to its HSR score and the food category as defined in [28]. Figure S2: Number of nuts and seeds containing products that bear each number of HSR stars, (by half units from 0.5 up to 5) according to the original HSR (S0) and scenarios S1, S2 and S3, attributed with category 2 thresholds. Table S1: Changes in the point scales for energy and SFA/total fats, in the scenario S3. Table S2: Data base of selected food products. Table S3: HSR scores and star numbers of each food product (original HSR and three scenarios S1, S2 and S3). Table S4: Variation in the number of stars derived from HSR scores, relatively to the original HSR, for scenarios S1, S2, and S3, for all foods, for foods containing les or more than 50% nuts and seeds and for each food category. Table S5: Variation in the HSR scores, relatively to the original HSR, for scenarios S1, S2, and S3, for all foods, for foods containing les or more than 50% nuts and seeds and for each food category. Table S6: Number and % of products with an improvement (more stars), deterioration (less stars) or no change in the HSR star number; for all products and for products with less than 50% nuts and seeds and those with 50% or more nuts and seeds.
