# Supplementary material for: Improved Recognition of the Nutrition and Health Benefits of Nuts and Seeds Within the Health Star Rating System
Source: Nutrients. 2025 Mar 29;17(7):1195. doi: 10.3390/nu17071195 (PMC11990889; doi:10.3390/nu17071195)
Supplement: Supplementary file 1 [file nutrients-17-01195-s001.zip › Supplementary figure S1.pptx]

## Slide 1
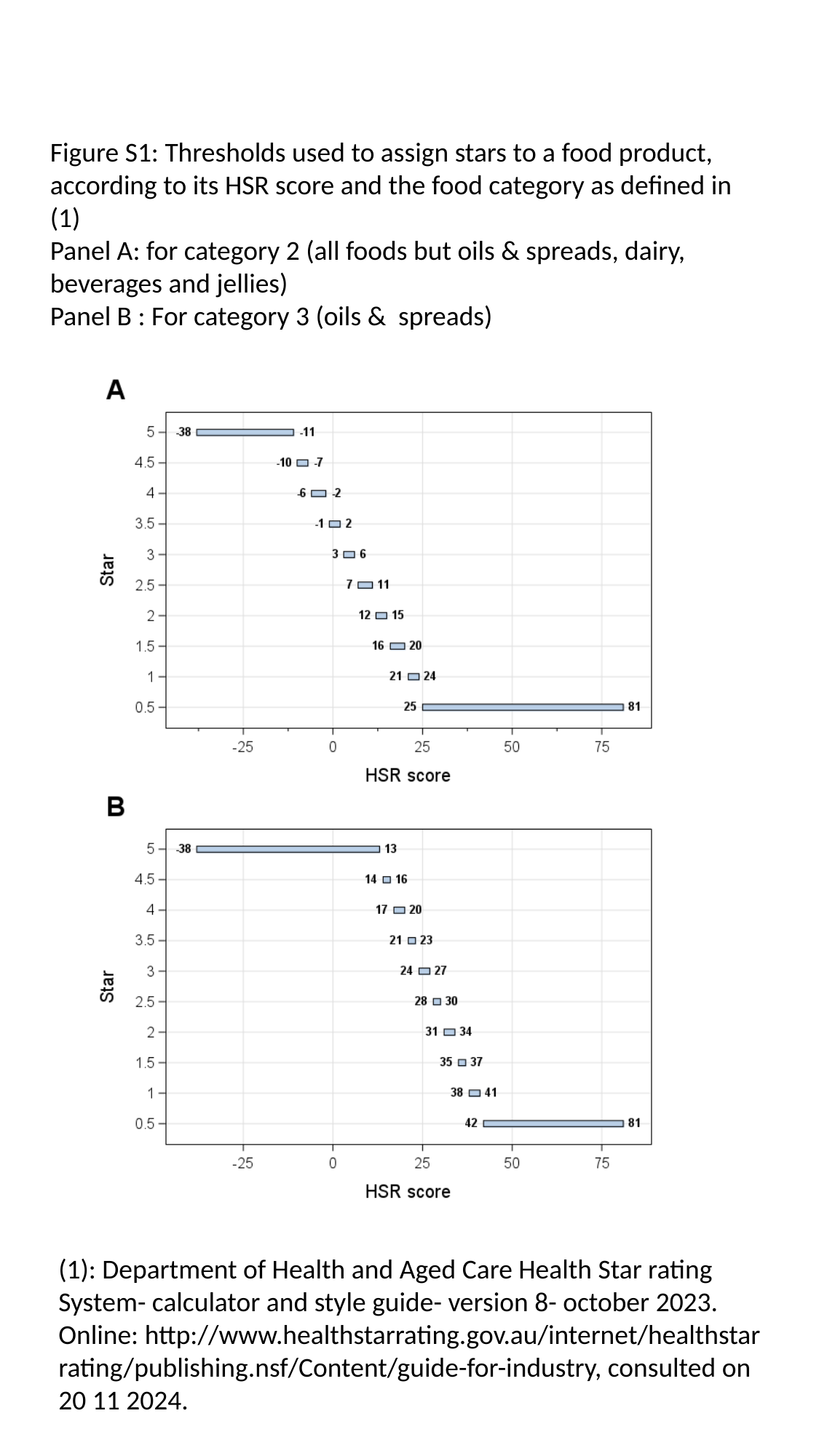

Figure S1: Thresholds used to assign stars to a food product, according to its HSR score and the food category as defined in (1)
Panel A: for category 2 (all foods but oils & spreads, dairy, beverages and jellies)
Panel B : For category 3 (oils & spreads)
(1): Department of Health and Aged Care Health Star rating System- calculator and style guide- version 8- october 2023. Online: http://www.healthstarrating.gov.au/internet/healthstarrating/publishing.nsf/Content/guide-for-industry, consulted on 20 11 2024.
