# Supplementary material for: Improved Recognition of the Nutrition and Health Benefits of Nuts and Seeds Within the Health Star Rating System
Source: Nutrients. 2025 Mar 29;17(7):1195. doi: 10.3390/nu17071195 (PMC11990889; doi:10.3390/nu17071195)
Supplement: Supplementary file 1 [file nutrients-17-01195-s001.zip › Supplementary figure S2.pptx]

## Slide 1
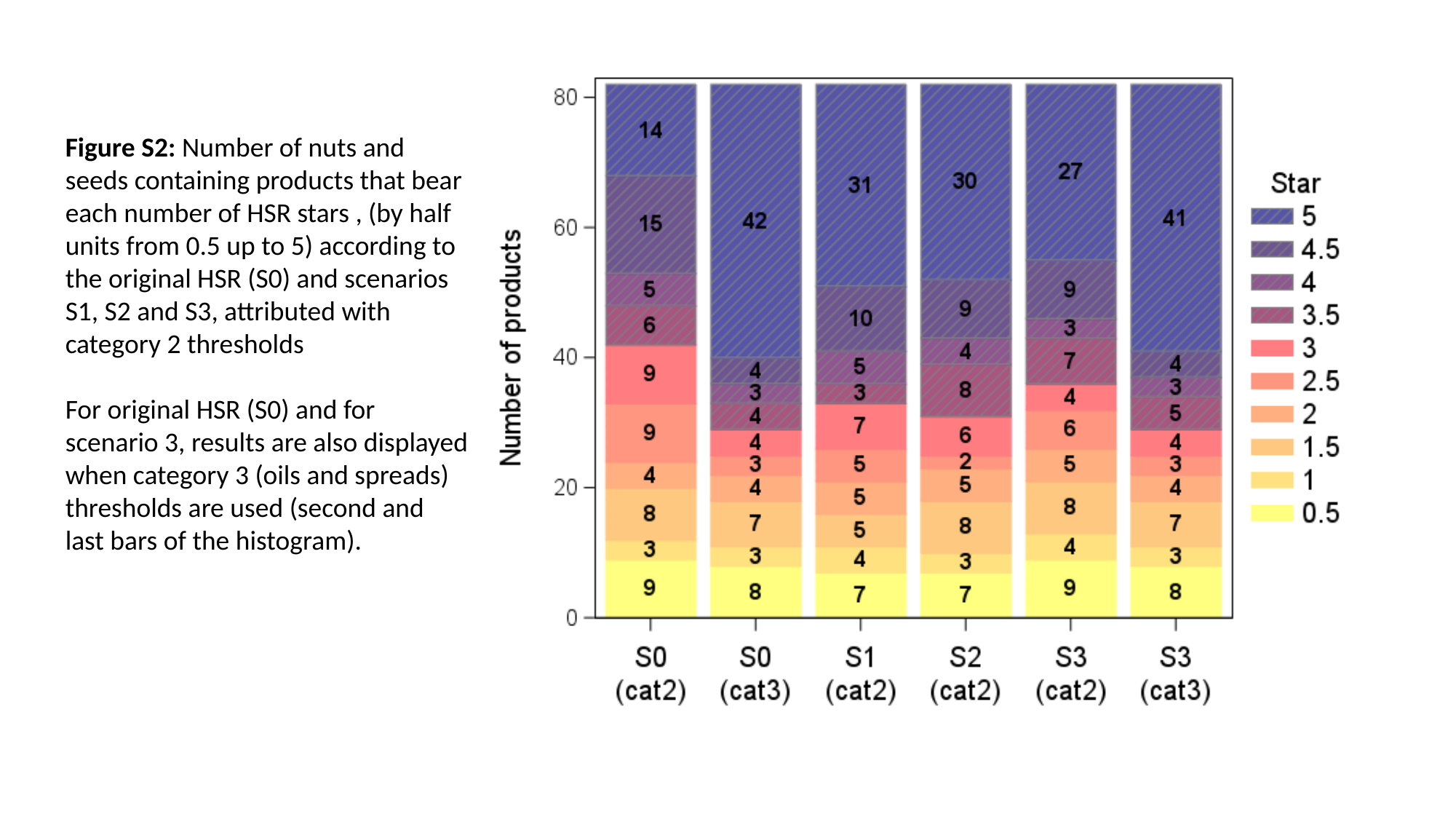

Figure S2: Number of nuts and seeds containing products that bear each number of HSR stars , (by half units from 0.5 up to 5) according to the original HSR (S0) and scenarios S1, S2 and S3, attributed with category 2 thresholds
For original HSR (S0) and for scenario 3, results are also displayed when category 3 (oils and spreads) thresholds are used (second and last bars of the histogram).
