# Supplementary material for: Improved Recognition of the Nutrition and Health Benefits of Nuts and Seeds Within the Health Star Rating System
Source: Nutrients. 2025 Mar 29;17(7):1195. doi: 10.3390/nu17071195 (PMC11990889; doi:10.3390/nu17071195)
Supplement: Supplementary file 1 [file nutrients-17-01195-s001.zip › Supplementary tables revised..pdf]

Supplementary tables

Table S1: Changes in the point scales for energy and SFA/total fats, in the scenario S3. Table displays the thresholds used in the latest revision of the French Nutri-Score and the adaptation made to points attribution in scenario S3, so that the whole original scale is used (i.e., 11 points for energy and 30 points for the ratio SFA/total fats).

Points	Energy from SFA, Kj/100g		Ratio SFA/total fats, %	
	Nutri-Score	HSR S3	Nutri-Score	HSR S3
0	<=120	**<=60**	<10	<6
1	>120	**>60**	<16	<8
2	>240	>120	<22	<10
3	>360	>240	<28	<12
4	>480	>360	<34	<14
5	>600	>480	<40	<16
6	>720	>600	<46	<18
7	>840	>720	<52	<20
8	>960	>840	<58	<22
9	>1080	>960	<64	<24
10	>1200	>1080	>=64	<26
11		>1200		<28
12				<30
13				<32
14				<34
15				<36
16				<38
17				<40
18				<42
19				<44
20				<46
21				<48
22				<50
23				<52
24				<54
25				<56
26				<58
27				<60
28				<62
29				<64
30				>=64

Table S2: data base of selected food products (see excel file)

Table S3: HSR scores and star numbers of each food product (original HSR and three scenarios S1, S2 and S3) (see excel file)

Table S4: Variation in the number of stars derived from HSR scores, relatively to the original HSR, for scenarios S1, S2, and S3, for all foods, for foods containing les or more than 50% nuts and seeds and for each food category.
Stars (*) indicate the level of statistical significance of score differences vs the original HSR *: P-value <0.005; **: P-value <0.0001.
Hashtags (#) indicate the level of statistical differences when comparing scores among products with more or less than 50% nuts and seeds #: P-value: 0.0002; ## P-value<0.0001
No statistic has been performed by food category, because of low sample size.

		S1 vs original HSR			S2 vs original HSR			S3 vs original HSR		
		Delta HSR star			Delta HSR star			Delta HSR star		
	**Sum**	**Mean**	**Min**	**Max**	**Mean**	**Min**	**Max**	**Mean**	**Min**	**Max**
**All foods**	82	0.41*	0.00	2.50	0.37*	0.00	1.50	0.17**	-2.00	1.50
Foods with less than 50% nuts and seeds	39	0.19#	0.00	1.50	0.18#	0.00	1.00	0.00##	0.00	0.00
Foods with 50% nuts and seeds or more	43	0.60	0.00	2.50	0.55	0.00	1.50	0.33	0.00	1.50
**Food categories**										
Breads	3	0.17	0.00	0.50	0.00	0.00	0.00	0.00	0.00	0.00
Breakfast cereals	8	0.19	0.00	1.00	0.31	0.00	1.00	0.00	0.00	0.00
Cakes	3	0.00	0.00	0.00	0.00	0.00	0.00	0.00	0.00	0.00
Cereal bars	15	0.83	0.00	2.00	0.80	0.00	1.50	0.37	0.00	1.50
Chocolate confectionary	6	0.08	0.00	0.50	0.17	0.00	0.50	0.00	0.00	0.00
Cookies	5	0.30	0.00	1.50	0.10	0.00	0.50	0.00	0.00	0.00
Ice creams	5	0.10	0.00	0.50	0.10	0.00	0.50	0.00	0.00	0.00
Non-chocolate confectionary	2	0.00	0.00	0.00	0.50	0.00	1.00	0.50	0.00	1.00
Nut butters	8	0.31	0.00	1.00	0.31	0.00	1.00	0.25	0.00	0.50
Nuts	13	0.65	0.00	2.50	0.42	0.00	1.00	0.08	-2.00	1.00
Seeds	5	0.00	0.00	0.00	0.00	0.00	0.00	0.00	0.00	0.00
mixed & coated/salted nuts	9	0.61	0.00	1.00	0.56	0.00	1.00	0.50	0.00	1.50

Table S5: Variation in the HSR scores, relatively to the original HSR, for scenarios S1, S2, and S3, for all foods, for foods containing les or more than 50% nuts and seeds and for each food category.
Stars (*) indicate the level of statistical significance of score differences vs the original HSR *: P-value <0.05; **: P-value <0.001.
Hashtags (#) indicate the level of statistical differences when comparing scores among products with more or less than 50% nuts and seeds #: P-value : 0.0002; ## P-value<0.0001
No statistic has been performed by food category, because of low sample size.

		**scenario**								
		**S1**			**S2**			**S3**		
	**nb**	**Delta HSR score**			**Delta HSR score**			**Delta HSR score**		
	**Sum**	**Mean**	**Min**	**Max**	**Mean**	**Min**	**Max**	**Mean**	**Min**	**Max**
**All**	82	-4.67**	-25.00	0.00	-4.5**7	-14.00	0.00	-2.83*	-13.00	16.00
Foods with less than 50% nuts and seeds	39	-1.79#	-14.00	0.00	-1.64##	-7.00	0.00	0.00##	0.00	0.00
Foods with 50% nuts and seeds or more	43	-7.28	-25.00	-1.00	-7.23	-14.00	-2.00	-5.40	-13.00	16.00
**Food category**										
Breads	3	-0.67	-1.00	0.00	-1.00	-2.00	0.00	0.00	0.00	0.00
Breakfast cereals	8	-1.13	-7.00	0.00	-2.25	-7.00	0.00	0.00	0.00	0.00
Cakes	3	-0.33	-1.00	0.00	0.00	0.00	0.00	0.00	0.00	0.00
Cereal bars	15	-7.33	-16.00	-3.00	-6.73	-14.00	-1.00	-3.27	-13.00	0.00
Chocolate confectionary	6	-1.50	-4.00	0.00	-2.33	-4.00	-1.00	-0.33	-2.00	0.00
Cookies	5	-3.60	-14.00	0.00	-1.40	-2.00	0.00	0.00	0.00	0.00
Ice creams	5	-0.60	-3.00	0.00	-0.40	-1.00	0.00	0.00	0.00	0.00
Non-chocolate confectionary	2	-0.50	-1.00	0.00	-4.50	-9.00	0.00	-4.50	-9.00	0.00
Nut butters	8	-5.13	-9.00	0.00	-5.63	-8.00	-1.00	-5.38	-9.00	0.00
Nuts	13	-8.69	-25.00	-2.00	-7.38	-9.00	-4.00	-4.85	-10.00	16.00
Seeds	5	-4.00	-7.00	-2.00	-5.80	-7.00	-5.00	-4.60	-7.00	-3.00
mixed & coated/salted nuts	9	-6.22	-9.00	-2.00	-5.67	-7.00	-3.00	-4.78	-10.00	0.00

Table S6: Number and % of products with an improvement (more stars), deterioration (less stars) or no change in the HSR star number; for all products and for products with less than 50% nuts and seeds and those with 50% or more nuts and seeds.

		S1 vs original HSR	S2 vs original HSR	S3 vs original HSR
		**N (%)**	**N (%)**	**N (%)**
**All products**				
	**DEGRADED**	.	.	2 (2)
	**IMPROVED**	39 (48)	42 (51)	22 (27)
	**UNCHANGED**	43 (52)	40 (49)	58 (71)
**Nuts & seeds content**				
**< 50% (N=39)**	**IMPROVED**	12 (31)	13 (33)	0 (0).
	**UNCHANGED**	27 (69)	26 (68)	39 (100)
**>=50% (N=43)**	**DEGRADED**	.	.	2 (5)
	**IMPROVED**	27 (63)	29 (67)	22 (51)
	**UNCHANGED**	16 (37)	14 (33)	19 (44)
